# Stress echocardiography for left ventricular diastolic dysfunction detection in patients with non-severe chronic obstructive pulmonary disease**: **a cross-sectional study

**DOI:** 10.3325/cmj.2019.60.449

**Published:** 2019-10

**Authors:** Radostina Vl. Cherneva, Mariana V. Gospodinova, Stefan V. Denchev, Rosen B. Petkov, Dimitar E. Kostadinov, Zheina Vl. Cherneva

**Affiliations:** 1 University Hospital for Respiratory Diseases “St. Sophia,” Sofia, Bulgaria; 2 University Hospital of the Ministry of Internal Affairs, Sofia, Bulgaria

## Abstract

**Aim:**

To assess whether the simultaneous performance of exercise stress echocardiography and cardio-pulmonary testing (ESE-CPET) may facilitate the timely diagnosis of subclinical left ventricular diastolic dysfunction (LVDD) in patients with non-severe chronic obstructive pulmonary disease (COPD), preserved left ventricular systolic function, and exertional dyspnea or exercise intolerance.

**Methods:**

This cross-sectional study, conducted between May 2017 and April 2018, involved 104 non-severe COPD patients with exertional dyspnea and preserved ejection fraction who underwent echocardiography before CPET and 1-2 minutes after peak exercise. Based on the peak E/e’ ratio, patients were divided into the group with stress-induced LVDD – E/e’>15 and the group without stress-induced LVDD. We assessed the association between LVDD and the following CPET variables: minute ventilation, peak oxygen uptake (VO_2_), ventilatory efficiency, heart rate reserve, and blood pressure.

**Results:**

During ESE-CPET, stress-induced LVDD occurred in 67/104 patients (64%). These patients had lower work load, peak VO_2_, O_2_ pulse, and minute ventilation (VE), and higher VE/VCO_2_ slope than patients without stress-induced LVDD (35.18 ± 10.4 vs 37.01 ± 11.11, *P* < 0.05). None of the CPET variables correlated with E/e’.

**Conclusion:**

Combined ESE-CPET may distinguish masked LVDD in patients with non-severe COPD with exertional dyspnea and preserved left ventricular systolic function. None of the CPET variables was a predictor for subclinical LVDD.

During the last decade, chronic obstructive pulmonary disease (COPD) has caused significant morbidity and mortality (1). COPD patients have greater than 2-fold excess risk of cardiovascular events and greater than 4-fold risk of developing chronic heart failure or coronary artery disease (2,3). Even patients with mild COPD have sub-clinical functional and structural changes, which, as airway obstruction gradually progresses, evolve to an overt disease (4). Therefore, an early detection and proper risk assessment are important for the control and prevention of cardiovascular co-morbidities in COPD.

Studies using magnetic resonance imaging established that patients with mild COPD without diseases, hypertension, or diabetes have arterial stiffness and myocardial fibrosis (5-8). Both pathologies are manifestations of the systemic effects of COPD and are a precondition for left ventricular diastolic dysfunction (LVDD) (9,10). In addition, chest wall hyperinflation and intra-thoracic pressure gradients may influence left ventricular (LV) pre-load and/or afterload (11). Thus, systemic and hemodynamic effects associated with the pathophysiological abnormalities of COPD are preconditions for LVDD.

Dyspnea and exercise intolerance are common symptoms of both COPD and heart failure with preserved ejection fraction (12,13). This is why there is need for cardio-respiratory parameters that discriminate between cardiac and respiratory nature of dyspnea or diminished physical activity (14,15).

There are no studies dealing with the problem of masked heart failure in COPD population. We hypothesized that the simultaneous performance of exercise stress echocardiography and cardio-pulmonary testing (ESE-CPX) may facilitate the timely diagnosis of subclinical LVDD in patients with non-severe COPD, preserved left ventricular systolic function (LVSF), and exertional dyspnea or exercise intolerance. The aims of the current study are to detect the frequency of exercise-induced LVDD in non-severe, non-symptomatic at rest COPD patients and to establish CPET parameters that can be applied as surrogate markers for masked LVDD.

## Materials and methods

### Patients and study protocol

**Figure 1:**
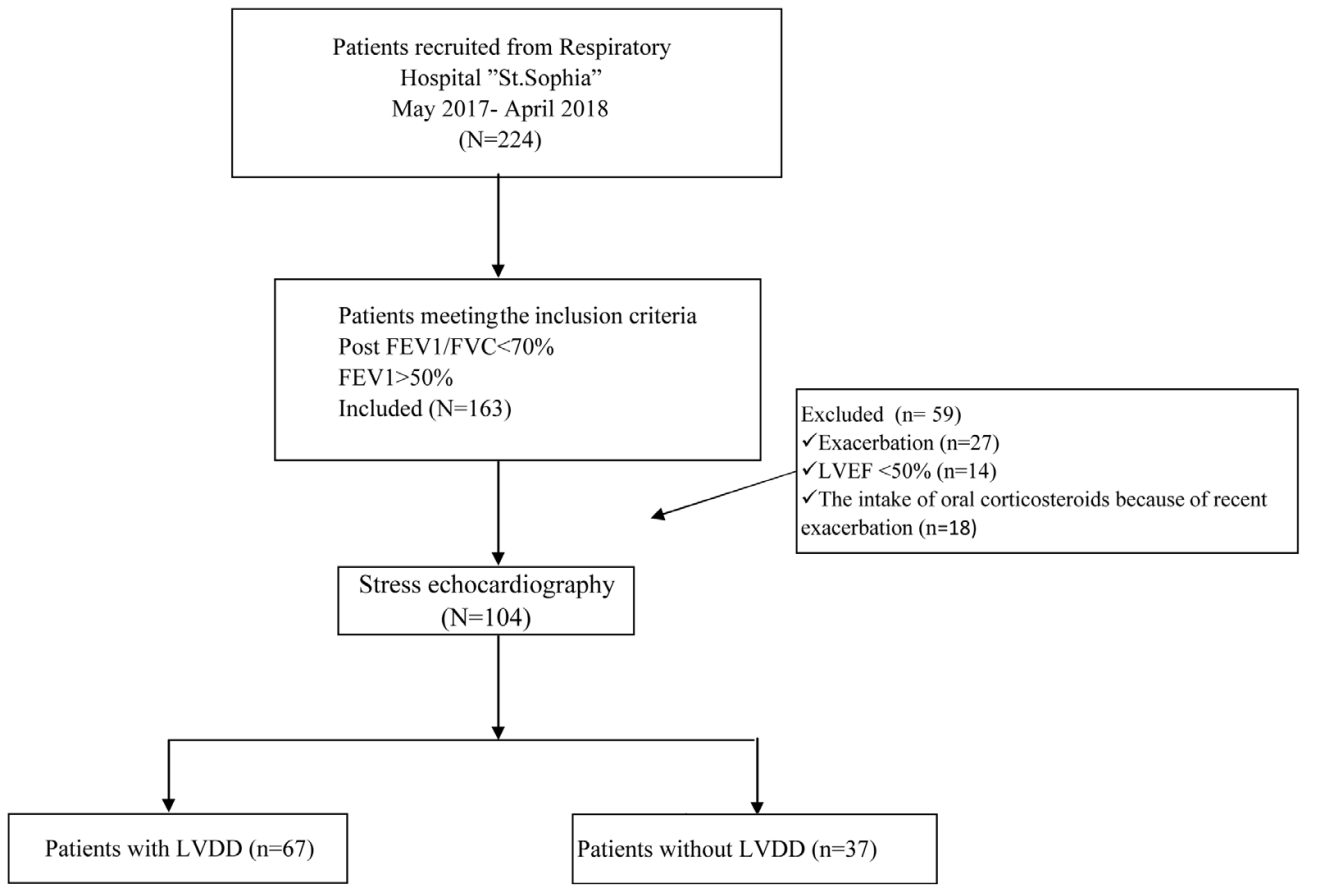
Study flowchart. FVC – forced vital capacity; FEV1 – forced expiratory volume 1; LVEF – left ventricular systolic function; LVDD left ventricular diastolic dysfunction.

This cross-sectional study involved 224 clinically stable outpatients diagnosed with COPD at the University Hospital for Respiratory Diseases “St. Sophia” in Sofia, Bulgaria. Only 163 of them met the inclusion criteria – FEO1 > 50% and lack of overt cardiovascular diseases. All patients had exertional dyspnea and preserved left ventricular systolic function. The following exclusion criteria were used: 1) LVEF<50%; 2) LVDD at rest more than first grade; 3) echocardiographic signs of systolic pulmonary arterial hypertension; 4) valvular heart disease; 5) documented cardiomyopathy; 6) severe uncontrolled hypertension (systolic blood pressure >180 mm Hg and diastolic blood pressure >90 mm Hg); 7) atrial fibrillation or malignant ventricular arrhythmia; 8) anemia; 9) cancer; 10) chronic kidney disease; 11) recent chest or abdominal surgery; 12) recent exacerbation (during the last three months); 13) recent change (during the last three months) in medical therapy. Based on these criteria, 59 patients were excluded, and 104 patients (64 men; mean age of 62.9 ± 7.5 years) were considered eligible (Figure 1). The recruitment period lasted between May 2017 and April 2018. The study was approved by the Committee of Ethics of Science Research, Medical University Sofia (protocol 5/12.03.2018). All the patients signed informed consent before entering the study.

Patients’ data included general demographic information, self-reported medical history, and concomitant medication. Demographic and clinical data are presented in Table 1.

**Table 1:** Clinical and demographic characteristics of patients with and without left ventricular diastolic dysfunction (LVDD)

Demographic data	Number (%) of patients		
**without stress -induced LVDD (N = 37)**	**with stress -induced LVDD (N = 67)**	***P***
Age (years), mean ± standard deviation	60.44 ± 7.72	64.16 ± 6.97	0.143*
Male, n	21:16	44:23	0.298*^ǂ^*
Current smokers	23 (62)	39 (58)	0.176*^ǂ^*
Former smokers	4 (11)	17 (25)	0.981*^ǂ^*
Non-smokers	10 (27)	11 (17)	0.375*^ǂ^*
Pack years, mean ± standard deviation	27.21 ± 6.33	33.79 ± 3.04	0.491*^ǂ^*
Body mass index (kg/m^2^), mean ± standard deviation	27.38 ± 4.58	26.65 ± 6.7	0.207^†^
**Clinical data**			
systolic blood pressure (mmHg), mean ± standard deviation	128.16 ± 9.68	128.17 ± 11.39	0.217^†^
diastolic blood pressure (mmHg), mean ± standard deviation	81.11 ± 5.54	82.31 ± 6.53	0.206^†^
**Discharge medication**			
inhaled β2 agonists	26(70)	67(100)	0.721*^ǂ^*
inhaled anticholinergic medications	17 (46)	38(57)	0.409*^ǂ^*
inhaled corticosteroids	18 (48)	50 (75)	0.031*^ǂ^*
angiotensin converting enzyme Inhibitors	22 (59)	54 (81)	0.026*^ǂ^*
β blockers	7 (19)	22 (33)	0.027*^ǂ^*
diuretics	25 (67)	52 (78)	0.043*^ǂ^*
**Medical history**			
diabetes	6 (16)	13 (19)	0.721*^ǂ^*
systemic hypertension	23 (62)	54 (81)	0.483*^ǂ^*
coronary artery disease	1 (2)	12 (18)	0.492*^ǂ^*

*Unpaired *t* test.

†Mann-Whitney U test.

ǂChi square test.

### Pulmonary function testing

All participants underwent preliminary clinical examination that included chest x-ray, spirometry, electrocardiogram, and echocardiography. The patients eligible for the study performed spirometry and exercise stress test. Both tests were performed on Vyntus (Carefusion, Baesweiler, Germany) following the manufacturer’s instructions. Spirometry was performed after bronchodilation test with the application of 400 mg salbutamol. Following the ERS guidelines, a post-bronchodilation ratio of FEV1/FVC<70% was used as a diagnostic criterion of COPD (16). Only patients with mild/moderate airway obstruction (FEV1 > 50%) were selected.

### Six-minute walk test

Six-minute walk test was performed in accordance with the ATS guidelines (17). It was done on a separate day after the initial visit and after the exercise stress test and stress echocardiography. Participants were instructed to walk the previously measured 30-m distance in a hospital corridor. SpO_2_, heart rate, and arterial blood pressure were obtained before and during the recovery period, 1 minute after the end of the test.

### Stress test protocol – cardio-pulmonary exercise testing (CPET)

After the clinical examination and spirometry, all the patients underwent symptom-limited incremental exercise stress test on a supine bicycle in accordance with the guidelines (18). Gas and flow sensors were calibrated before each test. Patients underwent standard electrocardiography through the whole exercise test and manual blood pressure measurements and heart rate recordings at the end of every stage.

A continuous RAMP protocol was applied – two minutes of unloaded pedaling (rest phase – 0W), a three-minute warm-up phase (20W), and the test phase including 20 W/2-min load increments. Patients were instructed to pedal with 60 rotations per minute.

Expiratory gases were analyzed by breath-by-breath analysis. Peak values of oxygen consumption, carbon dioxide production, and ventilatory efficiency (VE/VCO_2_) are presented as the highest 30-second average value obtained during the last stage of the exercise test. These values were used to estimate peak respiratory exchange ratio (RER). RER>1.00 was considered as a maximum effort. The modified Borg scale was applied for peak dyspnea evaluation. Heart rate recovery was defined as the difference between peak heart rate and heart rate during the first minute of recovery.

### Electrocardiography

Twelve-lead electrocardiography was performed during the four phases of the cardio-pulmonary test. The test was terminated because of exertion – exercise-limiting dyspnea, fatigue, claudication, or severe chest pain at patient’s request or because of clinical indications – emergence of repolarization ST-T changes, decrease in systolic blood pressure of more than 20 mm Hg, increase in systolic blood pressure of more than 220 mm Hg, or malignant arrhythmias.

### Echocardiography methods

Echocardiography included the generally applied approaches of M-mode and two-dimensional and Doppler echocardiography. Routine structural and hemodynamic indices of both chambers were measured following the guidelines (19). The systolic function of the left ventricle was defined by Simpson’s modified rule. The diastolic function of both ventricles was evaluated by the E/A ratio at rest (20) and tissue Doppler analysis as a more precise approach. The e’ value was defined as the average of the medial and lateral measurements of the mitral annulus. The peak of the average E/e’ ratio greater than 15 was considered as a marker for stress induced LVDD.

The dimensions of the right ventricle were assessed from the long-axis parasternal and apical four chamber view (21). TAPSE and S peak velocity were analyzed for RV systolic function evaluation. RVWT was measured in end-diastole. Systolic pulmonary arterial pressure was calculated by Bernoulli equation and by the acceleration time (AT) (22,23). Right atrium volume index (RAVI) was measured at end-systole by Simpson’s modified rule. Reference range for RAVI averages 25 ± 7 mL/m^2^ in men and 21 ± 6 mL/m^2^ in women. Stress induced RV diastolic dysfunction was considered if stress induced E/e’ ratio was higher than 6. All parameters were measured at end-expiration and in triplicate during different heart cycles.

### Statistical analysis

Descriptive statistics was used to summarize demographic and clinical data. Normality of distribution was tested with the Kolmogorov-Smirnov test. Continuous variables are expressed as mean values ± standard deviation, while categorical variables are expressed as proportions. Normally distributed variables were compared between the patients with and without LVDD with the *t* test, whereas non-normally variables were compared with the Mann-Whitney-U test. Categorical variables were compared with the χ^2^ test or Fisher exact test. The correlation between CPET parameters and the presence of LVDD was assessed by the Spearman’s rank correlation test. The level of significance was set at *P* < 0.05. The analyses were conducted in SPSS, v. 13.0 (SPSS Inc. Chicago, IL, USA).

## Results

Participants had a mean age of 62.9 ± 7.5 years and body mass index of 27.02 ± 6.3 kg/m^2^. According to the stress echocardiography results, they were divided into the group with stress-induced LVDD and the group without stress-induced LVDD. The groups did not differ in anthropometric, respiratory (Table 2), or other clinical characteristics. After ESE-CPX, 64% (67/104) of patients showed stress-induced LVDD (E/e’>15). Other LV and RV structural and functional parameters are shown in Table 3. Patients with stress-induced LVDD had a significant increase in systolic pulmonary arterial pressure (baseline 27.92 ± 2.97 mmHg vs stress induced 38.80 ± 3.83 mmHg, *P* < 0.0001) (Table 3). Non-severe COPD patients with stress-induced LVDD compared with non-severe COPD patients without stress-induced LVDD showed limited exercise capacity (Table 4), achieving lower load, VO_2_, and O_2_-pulse. They performed with significantly higher VE/VCO_2_ slope (35.18 ± 10.4 vs 37.01 ± 11.11, *P* = 0.032; Table 4). None of the CPET parameters correlated with stress induced LVDD.

**Table 2:** Respiratory parameters of patients with and without left ventricular diastolic dysfunction (LVDD)*

Parameter, mean ± standard deviation	Patients without stress-induced LVDD (N = 37)	Patients with stress-induced LVDD (N = 67)	*P^†^*
**Respiratory function**			
FVC, l/min	2.60 ± 0.93	2.38 ± 0.74	0.213
FEV 1, l/min	1.71 ± 0.66	1.47 ± 0.42	0.408
FEV1/FVC %	62.12 ± 9.46	53.87 ± 14.39	0.764
mMRC	1.55 ± 0.49	1.70 ± 0.79	0.891
**Acid-base balance**			
Ph	7.44 ± 0.03	7.43 ± 0.03	0.329
O_2_, mmHg	68.09 ± 10.44	67.48 ± 10.19	0.298
CO_2_, mmHg	35.96 ± 9.49	37.14 ± 8.03	0.275
BE, mmol/L	3.80 ± 5.63	2.39 ± 1.79	0.872
HCO_3_, mmol/L	21.73 ± 6.46	24.28 ± 3.48	0.328
Saturation %	94.15 ± 2.00	94.01 ± 2.27	0.763

*FVC – forced vital capacity; FEV1 – forced expiratory volume 1; mMRC – modified Medical Research Council; BE – base excess.

†Mann-Whitney U test.

**Table 3:** Echocardiographic characteristics of patients with and without left ventricular diastolic dysfunction (LVDD)*

Parameter, mean ± standard deviation	Patients without stress-induced LVDD (N = 37)	Patients with stress-induced LVDD (N = 67)	*P^†^*
**LV structural parameters**			
LVEF, %, Teicholz	62.69 ± 4.02	62.09 ± 6.34	0.653
LVEF, %, Simpson	63.05 ± 3.80	60.40 ± 6.44	0.016
TDD, mm	52.22 ± 5.16	50.98 ± 4.61	0.253
TSD, mm	33.94 ± 4.37	33.48 ± 4.72	0.275
TDV, mL	131.88 ± 30.18	122.44 ± 25.19	0.181
TSV, mL	49.63 ± 14.85	46.80 ± 16.32	0.187
Septum, mm	12.01 ± 1.01	12.00 ± 1.19	0.897
PW, mm	12.05 ± 0.94	12.03 ± 1.14	0.981
**LV functional parameters at rest**			
E/A ratio	0.94 ± 0.70	0.98 ± 0.32	0.420
E' average, cm/s	8.36 ± 1.47	9.62 ± 1.69	0.003
E/e' average ratio	7.30 ± 1.89	7.12 ± 2.59	0.736
**LV functional parameters after exercise stress test**			
E/A ratio	0.92 ± 0.68	1.79 ± 0.42	<0.0001
E' average, cm/s	10.55 ± 2.24	6.78 ± 0.90	<0.0001
E/e’ average	9.04 ± 1.89	17.18 ± 3.17	<0.0001
**RV structural parameters**			
RAVI, mL/m^2^	18.29 ± 2.65	23.04 ± 2.65	<0.0001
RVWT, mm	5.41 ± 1.08	6.53 ± 0.53	<0.0001
RV diameter parasternal, mm	28.02 ± 2.83	28.25 ± 2.25	0.687
RV basal, mm	36.30 ± 2.97	37.12 ± 3.25	0.095
RV med, mm	24.01 ± 3.43	27.24 ± 2.06	<0.0001
**RV functional parameters at rest**			
E/A ratio	0.92 ± 0.31	0.67 ± 0.14	<0.0001
E/e' average	4.72 ± 1.42	4.64 ± 1.01	0.064
TAPSE, mm	23.86 ± 2.82	22.39 ± 1.82	0.009
AT, ms	170.39 ± 12.08	169.59 ± 12.14	0.737
sPAP, mmHg	26.80 ± 2.42	27.92 ± 2.97	0.092
**RV functional parameters after exercise stress test**			
E/A ratio	1.39 ± 0.27	1.37 ± 0.21	0.830
E/e’ average	4.82 ± 1.6	9.60 ± 2.74	<0.0001
TAPSE, mm	21.25 ± 0.95	21.34 ± 1.85	0.324
AT, ms	163.33 ± 15.99	103.62 ± 16.04	<0.0001
sPAP, mmHg	31.86 ± 2.17	38.80 ± 3.83	<0.0001

*LVEF – left ventricular ejection fraction; TDD – telediastolic diameter; TSD – telesystolic diameter; TDV – tissue Doppler velocity; TSV – total stroke volume; PW – posterior wall thickness at end-diastole; RV – right ventricle; RAVI – right atrium volume index; RVWT – right ventricular wall thickness; TAPSE – tricuspid annular plane systolic excursion; SPAP –systolic pulmonary arterial pressure.

†Mann-Whitney U test.

**Table 4:** Cardio-pulmonary parameters of patients with and without left ventricular diastolic dysfunction (LVDD)*

Parameter, mean ± standard deviation	Patients without stress-induced LVDD (N = 37)	Patients with stress-induced LVDD (N = 67)	*P*
Peak load, W	82.75 ± 14.03	76.05 ± 13.34	0.041^‡^
Peak VE, L/min	59.8 ± 11.14	57.11 ± 10.07	0.148^‡^
Peak VO_2_, mL/min/kg	19.82 ± 4.27	18.97 ± 3.08	0.794^‡^
RER	1.03 ± 0.09	1.02 ± 0.08	0.808^‡^
BR<30%, estimated MVV	8%	11%	0.743^‡^
Peak HR, beats/min	131.6 ± 14.83	120.2 ± 16.86	0.626^‡^
PeakO_2_ pulse, mL/min/kg	9.97 ± 3 .1	8.68 ± 2.35	0.751^‡^
HRR	15.37 ± 8.22	14.84 ± 9.44	0.853^†^
Peak VE/VCO_2_ slope	35.18 ± 10.4	37.01 ± 11.1	0.032^‡^
Peak saturation, %	95 ± 2	94 ± 1	0.793^‡^

*LVDD – left ventricular diastolic dysfunction; VE – minute ventilation; VO_2_ – oxygen consumption; RER – respiratory exchange ratio; BR – breathing reserve; MVV – minute volume ventilation; HR – heart rate; HRR – heart rate reserve.

†Unpaired *t* test.

‡Mann-Whitney U test.

## Discussion

This study found 1) a high frequency (64%) of stress-induced LVDD in non-severe COPD patients with exertional dyspnea and preserved LV ejection fraction; 2) stress-induced elevation of systolic pulmonary arterial pressure (>35 mm Hg) and a reduced functional capacity, as measured by both CPET and 6-min walk, in participants with stress-induced LVDD; 3) difference in CPET variables between patients with and without stress-induced LVDD, but no correlation of these variable to stress E/e’. These results only partially confirm our hypothesis, as we expected that in addition to the high prevalence of stress-induced LVDD, some CPET parameters would predict LVDD. The reasons for the discrepancy may be the complex lung-heart interaction during exercise and multifactorial nature of LVDD.

COPD is a multi-systemic disease that remains a worldwide leading cause of disability (24,25). Compared with the general population, COPD patients bear a two to 5-fold increased risk of cardiovascular complications, with cardiovascular morbidity as the cause of death in a third of these patients (26). COPD is an independent predictor for vascular damage (27,28) and is associated with increased arterial stiffness and myocardial fibrosis, which may be present in COPD patients free of manifest cardiovascular diseases (11,28-32). Both pathological abnormalities are a precondition for a higher prevalence of LVDD in COPD patients (6,13). The link between LVDD and COPD is complex and is probably a result of various mechanisms – mechanical/functional (deterioration in FEV1, emphysema, hyperinflation) (5,11,33-35), biological (systemic inflammation, hypoxemia, endothelial dysfunction) (36-38), and neuro-humoral (excess sympathetic nerve activity) (39). Each of these may be a trigger in a certain phenotype of COPD patients (40-42).

Cardio-respiratory parameters may discriminate between the cardiac and respiratory nature of dyspnea/diminished physical activity and facilitate the early diagnosis of cardiovascular pathology in COPD. Our results, however, show that none of these parameters correlated with stress induced LVDD (E/e’>15). These results differ from data obtained in the general population, which had a significantly lower prevalence of exercise-induced LVDD. Nedeljkovic et al (43) performed ESE in 87 patients with exertional dyspnea, hypertension, and normal left ventricular function, 9.2% of whom demonstrated E/e’>15. In the study by Talal et al (44), among 87 patients with exertional dyspnea, 9% had E/A<0.75. Guazzi et al (45) reported an association between diastolic dysfunction (E/e’ ratio) and peak oxygen consumption, ventilatory efficiency, and heart rate recovery. In patients with normal echocardiography at rest, peak E/e’ ratio >15 correlated best to ventilatory efficiency (43). VE/VCO_2_ ratio as the best predictor of stress E/e’ was also confirmed in a diastolic heart failure group (45).

Since LVDD is associated with an increased risk of exacerbations and mortality, its timely diagnosis in non-severe COPD is important for disease management. As none of the clinical, ventilatory, cardio-pulmonary, and echocardiographic characteristics can predict stress-induced LVDD in non-severe COPD, the combined ESE can facilitate its early diagnosis.

Our study has the following limitations: 1) the relatively small sample size; 2) the lack of body plethysmography for the measurement of hyperinflation and the lack of diffusion capacity measurements, which are informative for the proper assessment of dyspnea; 3) measurements were performed at the end of expiration due to the huge pressure gradients accompanying the respiratory cycle in COPD patients; 4) measurements were performed in the early recovery period (approximately 2 min) after symptom-limited exercise. The timeline of pulmonary and intrathoracic pressures changes during the brief time interval from peak exercise to their measurement in early recovery is not well known, leading to possible underestimation.

In non-severe COPD patients with preserved LV systolic function, the combined ESE-CPX test can reliably identify stress-induced LVDD, the early diagnosis and proper management of which may facilitate the prevention of cardiovascular complications. Although none of the routine clinical and CPET parameters predicted increased E/e’, the high prevalence of exercise-induced LVDD in these patients indicates the need for performing stress echocardiography. 
